# Household food insecurity and associated factors in the Northeast of Iran: a cross-sectional study: Household food security in Northern Iran

**DOI:** 10.1186/s40795-022-00665-x

**Published:** 2023-01-03

**Authors:** Mohammad Reza Honarvar, Masoomeh Gholami, Zahra Abdollahi, Farzaneh Sadeghi Ghotbabadi, Farhad Lashkarboluki, Majid Najafzadeh, Mohsen Mansouri, Gholamreza Veghari, Nasser Behnampour

**Affiliations:** 1 Health Management and Social Development Research Center, Golestan University of Medical Sciences, Gorgan, Iran; 2 Secretariat of Supreme Council for Health and Food Security-Ministry of Health & Medical Education, Tehran, Iran; 3 Ministry of Health and Medical Education Deputy for health Nutrition Department Tehran, Tehran, Iran; 4 Department of Statistics and Information Technology, Golestan University of Medical Science, Gorgan, Iran; 5 Ischemic Disorders Research Center, Golestan University of Medical Sciences, Gorgan, Iran; 6 Department of Biostatistics and Epidemiology, Health Management and Social Development Research Center, Golestan University of Medical Sciences, Gorgan, Iran

**Keywords:** Food insecurity, HFIAS, GIS, Household

## Abstract

**Background:**

Food Insecurity (FI) is a global health concern. For the first time, this study evaluated households’ food insecurity and factors related to it in Golestan province, North of Iran.

**Methods:**

This cross-sectional study was conducted on 5129 randomly selected households in the Golestan Province in 2016. Sociodemographic characteristics, including age, ethnicity, household size, education level, and occupation status, were collected via interview. The prevalence and severity of food insecurity were identified by the Household Food Insecurity Access Scale (HFIAS), whose scores are between 0 and 27, with larger values indicating more severe food insecurity. The prevalence of food insecurity based on the geographical area was presented using GIS.

**Results:**

Out of 5129 households, 2216 (43.21%) had food security, and 2913 (56.79%) households had food insecurity, with a Mean ± SD HFIAS score of 4.86 ± 5.95. Out of 2913 households with FI, 1526 (52.39%), 956 (32.82%), and 431 (14.79%) had mild, moderate, and severe food insecurity, respectively. Among 14 regions of the province, three regions had the most cases of food insecurity. Food insecurity (moderate or severe) was significantly associated with mothers as the household head (adjusted OR = 1.67, 95% CI: 1.03–2.70) and lower education level of the household head.

**Conclusion:**

The prevalence of household food insecurity in the Golestan Province is higher than the national average. Factors such as literacy, employment status, and gender of the household head can be significantly associated with food insecurity.

**Supplementary Information:**

The online version contains supplementary material available at 10.1186/s40795-022-00665-x.

## Introduction

Household food insecurity is characterized by family members’ insufficient access to food due to a lack of resources or money [1, 2]. In fact, availability, access, utilization, and stability have been regarded as the pillars of food security [3]. Food insecurity typically affects socio-economically disadvantaged people and is associated with lower productivity, lower academic achievement [3, 4], and behavioral, educational, emotional, and health problems [5].

As a key indicator of nutritional status, household food security should be monitored based on the nutritional status of the overall population [6]. According to the Food and Agriculture Organization of the United Nations (FAO), more than 2 billion people worldwide suffer from food insecurity, particularly those in developing countries [7]. There is considerable variation in the prevalence of food insecurity, even at the country level [8]. In 2017, an estimated 11.8% of American households experienced food insecurity at least some of the time during the year. This means family members lacked access to enough food to maintain a healthy and active lifestyle [1]. These estimates are 21% higher in households with children [5, 9]. It was reported that more than 12% of Canadians were food insecure in 2017–18, which might have increased with the COVID-19 pandemic [10]. In different parts of Iran, food insecurity varies considerably between 11% in Tehran and 44% in Yazd [11]. Golestan province, located in the north of Iran, accepts immigrants, especially from Sistan and Baluchestan, with a relatively high ethnic diversity. In previous national surveys, it was among the relatively safe food provinces, but we do not have reliable information about the distribution of food insecurity between its rural and urban areas [12].

Determining the prevalence of food insecurity and related factors is essential for appropriate policy-making to reduce or control this issue [11]. Using tools that determine the spatial distribution of food insecurity helps to better analyze the factors related to food insecurity. In this regard, utilizing the geographic information system (GIS) as a novel means of analyzing and displaying quantitative spatial data is beneficial in identifying the most vulnerable parts of society regarding food insecurity [13]. This study is the first study that determines the prevalence of household food insecurity based on the geographical location in Golestan province. The Golestan SAMAT system (abbreviation of food and nutrition security system in Farsi) is designed so that household food insecurity information based on the place of residence of people in the geography of Golestan province is recorded in it [14]. Using simple and low-cost location-based tools such as the SAMAT system can help to accurately identify food insecurity hotspots at the city or village level in the province and make subsequent decisions to implement local community-based interventions. Therefore, this study was conducted to estimate the prevalence of food insecurity and its related factors and geographical distribution in the north of Iran in 2016.

## Methods

### Study setting

This cross-sectional study was carried out from June 5, 2016 to November 20, 2016 on 5129 households in Golestan, Iran. According to the 2016 census, the population of this province is estimated to be 1,868,819 people in 14 cities and 550,249 households.

Based on a study that reported the proportion of households with degrees of insecurity equal to 0.61 [15], the sample size for a limited population at the confidence level of 0.95, and the maximum error of 0.05 per 40,000 households was determined as 362 households.

The population of the province’s cities was divided into 14 regions with a population of 40,000 households then the required number of samples was selected from each region.

Therefore, the total sample size was determined to be 5068 households. Considering the 5% drop, the starting sample size was 5334 households.

Information on households was obtained from local healthcare databases. The households studied in this research were selected using systematic random sampling. So first, the list of household numbers was prepared from the local healthcare database, and then the first sample was randomly selected from the first 50 households. In the next step, other households were selected at a fixed distance from the first household. This sampling method was done separately in each city. Each household was then given a specific number, and the selection of households was carried out systematically according to the required sample size. Inclusion criteria for the head of household included age of ≥15 years, being a resident in the province for at least 6 months, having no mental disability, and willingness to participate in the study. Two hundred five households out of 5334 (3.8%) did not participate in the study or gave incomplete answers, which were not significantly different in terms of variables such as the gender of the head of the household, literacy level, and income. The respondent was preferably the mother or the head of the household. One hundred fifty-two households (3%) were single-person households, 31 were male, and 121 were female. The sampling framework and its implementation method aimed to generalize the results to each region and province. The Ethics Committee of Golestan University of Medical Sciences approved this study with the ethical code IR.GOUMS.REC.1394.265.

### Demographic and socioeconomic data

The demographic and socioeconomic characteristics of the households, including the age of the mother, ethnicity, household size, education level of the father and mother, and the occupational status of the mother and father, were collected by interviewing the head of each household. The geographical coordinates of each household were also recorded using a Garmin 60 GPS (made in Taiwan).

### Household food insecurity

This study assessed household food insecurity using the Household Food Insecurity Access Scale (HFIAS). The validity and reliability of the scale have been confirmed in the Iranian population [16]. The HFIAS consists of nine items assessing household food insecurity experiences within the previous 4 weeks. Three indicators of household food insecurity include the HFIAS score, the severity of food insecurity, and the overall prevalence of food insecurity calculated using responses to these nine conditions. According to the frequency of occurrence (rarely, sometimes, or often), affirmative responses are scored from 0 to 3. Total scores of 0–1, 2–7, 8–14, and 15–27 indicated food security, mild food insecurity, moderate food insecurity, and severe food insecurity in the household, respectively. The household was defined as “food secure” if the participant answered “no” to all items (e.g., HFIAS score equal to zero).

This system helps local authorities identify food insecurity sites and colonies to implement appropriate interventions. The data of this research and the geographical location of the households were recorded by Garmin 60 GPS in the SAMAT system.

### Statistical analysis

The R 4.1.2 software was recruited for data analysis. Descriptive statistics in the form of mean, standard deviation (SD), and percentage are used for expressing data. The Association of sociodemographic characteristics with food insecurity was assessed using univariate and multiple binary logistic regression models. The sociodemographic characteristics significantly related (*p* < 0.05) to food insecurity in the univariate model were included in a subsequent analysis using the multiple binary logistic regression model. We also used the GIS and inverse distance weighting interpolation to estimate the severity of food insecurity in different areas by displaying the intensity in a graduated color map. The higher intensity of food insecurity, the deeper the purple spectrum.

## Results

According to the results, out of 5129 surveyed households, 2913 reported a degree of food insecurity. Most of the household heads were fathers (90.35%). The majority of respondents were of Fars ethnicity (40.37%), the mean age was 42.80 ± 13.81, and the family size was 3.96 ± 1.48 (Table 1). Most fathers had primary or middle education (38.92%), and most were laborers or self-employed (56.56%). Mothers were mainly housewives (92.37%) and had a primary or middle education level (32.47%). More information is in the supplementary.

**Table 1:** Sociodemographic characteristics of the participants

Characteristics	Number (%)
**Father’s educational level**	
Illiterate	728 (15.05)
Basic literacy	620 (12.82)
Primary or middle school	1882 (38.92)
High school	966 (19.98)
University degree	640 (13.23)
**Mother’s education level**	
Illiterate	1322 (26.03)
Basic literacy	859 (16.92)
Primary or middle school	1649 (32.47)
High school	852 (16.78)
University degree	396 (7.80)
**Ethnicity**	
Fars	2045 (40.37)
Turkmen	1900 (37.50)
Sistani	682 (13.46)
Baluchi	198 (3.91)
Other	241 (4.76)
**Father’s occupational status**	
Unemployed	273 (5.65)
Farmer/rancher	779 (16.13)
Laborer	1254 (25.96)
Public sector employees	481 (9.96)
Self-employed	1478 (30.60)
Jobless/retired	565 (11.70)
**Mother’s occupational status**	
Housewife	4682 (92.37)
Employed	387 (7.63)
**Head of households**	
Father	4624 (90.35)
Mother	401 (7.84)
Other	93 (1.82)
Age, Mean ± SD	42.80 ± 13.81
Family size, Mean ± SD	3.96 ± 1.48

**Fig. 1:**
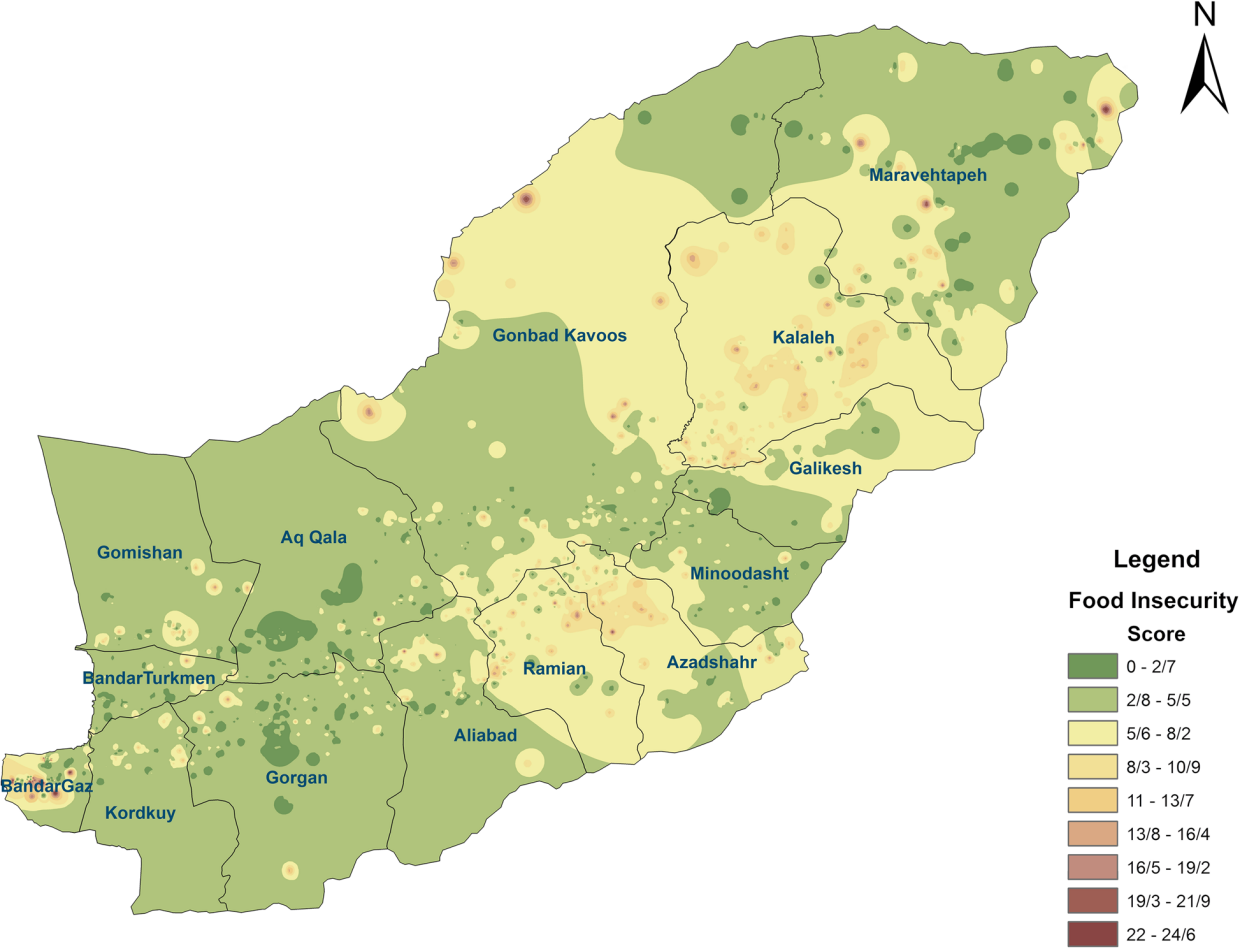
Geographical Food insecurity distribution in Golestan province, Iran, 2016

Among 14 districts of the province, Ramyan, Azadshahr, and Kalaleh had the most cases of food insecurity (Fig. 1). Parts of Gonbad-e-Kavus, Maraveh Tappeh, and Bandar Gaz cities showed severe or moderate food insecurity, but there were also food security points in the same cities. All cities showed scattered areas of food insecurity, but the distribution of food insecurity in the province was heterogeneous. Food insecurity was higher in the eastern and southern half of the province than in the western and northern half.

Severe food insecurity prevalence was 8.40% (95% confidence interval [CI] 7.67–9.19%) with a Mean ± SD HFIAS score of 4.86 ± 5.95. Out of 5129 households, 2216 (43.21%), 1526 (29.75%), 956 (18.64%), and 431 (8.4%) had food security, mild, moderate, and severe food insecurity, respectively.

According to the results of unadjusted analyses, food insecurity was significantly associated with ethnicity, the educational level of the father, the educational level of the mother, the father’s occupational status, the mother’s occupational status, and the head of the family (Table 2). Based on the multiple logistic regression analysis results, the low education level of fathers and mothers was significantly associated with the risk of food insecurity. Housewives are twice as likely to suffer from food insecurity as employed mothers (adjusted OR = 2.19, 95% CI: 1.78–2.70). A household headed by a mother was significantly more likely to suffer from food insecurity (adjusted OR = 1.67, 95% CI: 1.03–2.70). Subjects of Sistani (adjusted OR = 1.48, 95% CI: 1.19–1.85) and Baluchi (adjusted 2.75, 95% CI: 1.69–4.46) ethnicities had a significantly higher chance of experiencing food insecurity than the Fars counterparts (Table 2).

**Table 2:** Crude and adjusted odds ratios for factors associated with food insecurity among households in the Golestan Province, Iran (*n* = 5129)

***Characteristic***	***Unadjusted*** ***OR (95% CI)***	***P-value***	***adjusted*** ***OR (95% CI)***	***P-value***	***P-value***
***Father’s educational level***					
*Illiterate*	6.42 (5.07–8.14)	< 0.001	2.62 (1.88–3.64)	< 0.001	0.122^a^
*Basic literacy*	6.38 (4.98–8.17)	< 0.001	2.82 (2.05–3.88)	< 0.001	
*Primary or middle school*	4.45 (3.67–5.39)	< 0.001	1.99 (1.55–2.55)	< 0.001	
*High school*	1.96 (1.60–2.42)	< 0.001	1.20 (0.94–1.53)	0.13	
*University degree*	Ref		Ref		
***Mother’s education level***					
*Illiterate*	5.20 (4.08–6.62)	< 0.001	1.60 (1.15–2.24)	0.005	0.181^a^
*Basic literacy*	5.21 (4.03–6.74)	< 0.001	1.88 (1.36–2.61)	< 0.001	
*Primary or middle school*	3.81 (3.01–4.81)	< 0.001	1.57 (1.17–2.10)	0.002	
*High school*	2.04 (1.59–2.62)	< 0.001	1.29 (0.97–1.73)	0.07	
*University degree*	Ref		Ref		
***Ethnicity***					
*Fars*	Ref		Ref		
*Turkmen*	1.26 (1.11–1.43)	< 0.001	1.00 (0.86–1.16)	0.95	
*Sistani*	2.31 (1.90–2.80)	< 0.001	1.48 (1.19–1.85)	< 0.001	
*Baluchi*	5.56 (3.59–8.60)	< 0.001	2.75 (1.69–4.46)	< 0.001	
*Other*	1.46 (1.10–1.93)	0.008	1.75 (1.28–2.40)	< 0.001	
***Father’s occupational status***					
*Laborer*	Ref		Ref		
*Unemployed*	1.22 (0.867–1.721)	0.253	–		
*Farmer/rancher*	0.29 (0.24–0.35)	< 0.0001	0.23 (0.15–0.34)	< 0.001	
*Public sector employees*	0.07 (0.05–0.09)	< 0.0001	0.18 (0.12–0.29)	< 0.001	
*Self-employed*	0.25 (0.21–0.29)	< 0.0001	0.34 (0.23–0.50)	< 0.001	
*Jobless/retired*	0.18 (0.15–0.23)	< 0.0001	0.20 (0.13–0.30)	< 0.001	
***Mother’s occupational status***					
*Housewife*	2.19 (1.78–2.70)	< 0.001	1.53 (1.16–2.02)	0.002	
*Employed*	Ref		Ref		
***Head of household***					
*Father*	Ref		Ref		
*Mother*	2.09 (1.64–2.65)	< 0.001	1.67 (1.03–2.70)	0.03	
*Other*	1.78 (1.12–2.85)	0.01	1.65 (0.82–3.34)	0.15	

^a^Mann-Kendall trend test

## Discussion

In the Golestan Province, northwest of Iran, this is the first study to investigate household food insecurity. Out of 5129 households surveyed in the present study, 2913 (56.8%) households experienced some food insecurity. Severe food insecurity was observed in 8.4% of the households, particularly in Azadshahr, Ramyan, and Kalaleh. These three regions are similar to other regions in terms of age and proportion of heads of households, but in terms of literacy level, they were lower than other regions. Regarding the male head of the household, the percentage of labor jobs was higher, and the rate of (governmental) employees was lower than in other regions. In the three regions, the ratio of Sistani was two times, and the ratio of Baloch was four times more than in other regions. Additionally, 29.75, 18.64, and 8.4% of the population reported mild, moderate, or severe food insecurity, respectively. Factors such as the father’s or mother’s low literacy, the father’s or mother’s occupation, ethnicity, and the number of family members were related to food insecurity in Golestan province. As we mentioned earlier, the Sistani and Baluchi were mainly a migrant population who migrated from Sistan and Baluchistan province to Golestan many years ago due to famine. They often settled in certain geographical areas of the province and often had low-income labor jobs. We think that in addition to policymaking at the national and provincial levels, to reduce the factors related to household food insecurity, delegation more authority to local managers to implement local interventions based on local facilities, capabilities and conditions, including specific interventions to empower and employment of more vulnerable groups such as Sistani and Baluchi can help reduce food insecurity.

Researchers reported 28.6% mild, 14.9% moderate, and 6.0% severe food insecurity in Iran in a meta-analysis of experiences/perceptions [17]. Also, Payab et al. reported that 50.2% of mothers with primary school children in Ray (a County of Tehran) suffer from food insecurity [18]. In other studies, different prevalences of food insecurity have been reported in different parts of Iran. For example, the majority of food insecurity in different parts of Iran was as 37.8% in Tehran, 34.3% in Kerman, 58.8% in Zahedan, and 50.7% in rural areas of Gilan [19–22]. According to the FAO, about two billion people around the world are dealing with moderate or severe food insecurity [23]. Food insecurity affects developing and developed countries and could be as low as about 10% in the USA to as high as 100% in Sudan [23, 24]. The prevalence of moderate or severe food insecurity worldwide has increased slightly from 22.6 to 26.6% from 2014 to 2019. This prevalence in Golestan province is almost in the same range as in other parts of Iran, but by comparing food insecurity in the urban and even rural areas (Fig. 1), the areas with more severe food insecurity are identified. It seems that in addition to determining policies to reduce food insecurity at the provincial level, local interventions based on the potential local capabilities of each region should be designed and implemented in cities or villages [25]. These statements demonstrate the importance of more accurate local assessments to understand the factors influencing food insecurity and designing local interventions.

In our study, food insecurity was significantly associated with gender, literacy, household size, and the head of household employment status. The relationship between food insecurity with being female as head of the household in the studies of Gilan and Kerman, with the education level of the head of the household in the studies of Tabriz and Kerman, with the number of household members in the studies of Kerman, Tehran, and Tabriz and with the employment status of the head of the household in the studies of Kerman and Tehran were confirmed [19, 20, 22, 26, 27]. A cross-sectional study of food insecurity and predictors in the elderly of the United States found depression severity, financial instability, and a nutritional support package to be significantly associated with food insecurity [14]. Consistent with other studies, with the increase in the number of household members, especially those without income, i.e., children and the elderly, the need for adequate food increases, which in case of insufficient supply, increases the risk of malnutrition and food insecurity [28–30]. Our study showed the importance of parental employment in reducing food insecurity. This relationship has been emphasized in other studies in Iran (Tabriz, Kerman, Qazvin). Having a job increases the household’s economic access to food resources [20, 27, 29]. In our research, there is an inverse relationship between the mother or father’s education level and the state of food insecurity. Other studies have also shown this inverse relationship [31, 32]. Literate and educated parents are more likely to have better nutritional knowledge, which affects the household’s food intake.

Since parents, especially mothers, are the main decision-makers of household food consumption, promoting maternal health literacy and nutritional literacy is essential for proper family nutrition [31]. When access to food is restricted, household members inevitably eat less or do not receive some of their meals. In the study of Kohhande et al., the results of dietary diversity showed little variation in the dietary status of households. Due to the reduction of access to diverse food sources and the subsequent development of malnutrition, especially in micronutrients, this situation may indicate an increased likelihood of food insecurity in households. Families with more educated parents, especially more educated women, benefit from better nutrition and are less prone to malnutrition [32]. Policymakers need to increase the literacy of household members, especially mothers, to reduce food insecurity in the long term. In addition, training the household head, especially the mother, on choosing suitable foods according to the household’s income level can help reduce food insecurity in the short term. The country’s Supreme Health Council has compiled and approved the national document on nutrition and food security in Iran, in which programs such as education, women’s empowerment, nutritional support, and cash subsidies are provided to support food-insecure households [12].

In the present study, we used the SAMAT system for GIS-mapping and identification of major food-insecure areas in the Golestan Province [14]. Moore et al. used GIS to study the relationships between grocery store access and diet quality across states in the US. In participants who lived without supermarkets nearby, the likelihood of eating healthy was 25 to 46% lower than in participants who lived near supermarkets [33]. Kim et al. used GIS to reveal a strong relationship between the accessibility of green space and obesity predictors [34]. In another study, GIS-assisted and multivariate logistic regression analyses demonstrated that increasing obesity rates were associated with geographic proximity to fast-food restaurants (OR ≥ 1.2), and a decrease in obesity rates was related to living in neighborhoods with green spaces [35]. Indeed, GIS can be employed to better manage potential food resources, thereby reducing food insecurity [36]. Previous research in Nevada, USA, used GIS to investigate relations between social determinants of health and food insecurity. The study demonstrated that students living in catchment areas with higher food insecurity rates were more likely to be absent from schools. Although free meals have been provided for poor students in certain schools, the high absenteeism rates further hinder the food intake of poor students [37]. Climate-related conditions such as drought, flood, land degradation, and vegetation that influence food production and accessibility can also be investigated using GIS [38]. Household location and geographic inequalities can influence the relationship between socio-economic factors and food security. These results indicate that the prevalence and severity of food insecurity in the Golestan Province are higher than the national average. The existence of food insecurity in more than half of the households in the province requires the attention of the local government and support for interventions to reduce the food insecurity of households. Since food insecurity points are marked on the GIS map of the province, it is possible to conduct more detailed local surveys in known food insecurity areas. In all regions of the province, there were food-insecure colonies in both villages and urban areas. Identifying these hot points can help local authorities take appropriate and accurate measures to address food insecurity in the province. The location of households is a crucial factor determining the socio-economic factors and food security relations in urban areas of Iran [39]. Government and food insecurity-related institutions can promote and support community-based interventions in these areas.

Although this study was conducted with a large sample size and a large provincial scale, which can provide appropriate estimates of food insecurity at the urban and even rural levels, in expressing the limitations of this research, it should be noted that access to some of the families located in remote geographical areas and locating the GPS of those families during the implementation caused problems that we tried to solve with the help of the technical team. Some families were not present during the interviews, which were undertaken according to the neighboring neighbors’ executive protocol. This means that if it was impossible to reach the household after three visits, the first neighboring household was questioned. Also, due to the limitations of cross-sectional studies, we may not be able to show the lead-lag relationship between variables and food insecurity.

## Conclusion

The prevalence of household food insecurity in the Golestan Province is higher than the national average. Factors such as literacy, employment status, and gender of the household head can be significantly related to food insecurity. Poor economic status, illiteracy of the household head, having a female as head of the household, and household size are some of the influential factors in the household food program. It is essential to improve the food security of households. Local and central governments, governmental and non-governmental organizations, and even charities should help improve households’ livelihood and food security by implementing community-based interventions. It is clear that while food aid packages are helpful in some cases, the policies adopted to improve food security are ineffective [40].

## Supplementary Information

**Additional file 1.**

## Data Availability

The datasets used and/or analysed during the current study available from the corresponding author on reasonable request**.**
