# Supplementary material for: Household food insecurity and associated factors in the Northeast of Iran: a cross-sectional study: Household food security in Northern Iran
Source: BMC Nutr. 2023 Jan 3;9:5. doi: 10.1186/s40795-022-00665-x (PMC9807972; doi:10.1186/s40795-022-00665-x)
Supplement: Supplementary file 1 — Additional file 1. [file 40795_2022_665_MOESM1_ESM.docx]

| **Region * ethnicity Crosstabulation** | | | | | | | | |
| --- | --- | --- | --- | --- | --- | --- | --- | --- |
|  | | | ethnicity | | | | | Total |
|  |  |  | Fars | Turkmen | sistani | baluchi | other |  |
| Region | 3 region | Count | 302 | 245 | 206 | 89 | 11 | 853 |
|  |  | % within Region | 35.4% | 28.7% | 24.2% | 10.4% | 1.3% | 100.0% |
|  | others region | Count | 1743 | 1655 | 476 | 109 | 230 | 4213 |
|  |  | % within Region | 41.4% | 39.3% | 11.3% | 2.6% | 5.5% | 100.0% |
| Total | | Count | 2045 | 1900 | 682 | 198 | 241 | 5066 |
|  |  | % within Region | 40.4% | 37.5% | 13.5% | 3.9% | 4.8% | 100.0% |

| **Region * father.job Crosstabulation** | | | | | | | | | |
| --- | --- | --- | --- | --- | --- | --- | --- | --- | --- |
|  | | | father.job | | | | | | Total |
|  |  |  | Unemployed | Farmer/rancher | Laborer | Public sector employees | Self-employed | Jobless/retired |  |
| Region | 3 region | Count | 34 | 154 | 324 | 70 | 186 | 44 | 812 |
|  |  | % within Region | 4.2% | 19.0% | 39.9% | 8.6% | 22.9% | 5.4% | 100.0% |
|  | others region | Count | 239 | 625 | 930 | 411 | 1292 | 521 | 4018 |
|  |  | % within Region | 5.9% | 15.6% | 23.1% | 10.2% | 32.2% | 13.0% | 100.0% |
| Total | | Count | 273 | 779 | 1254 | 481 | 1478 | 565 | 4830 |
|  |  | % within Region | 5.7% | 16.1% | 26.0% | 10.0% | 30.6% | 11.7% | 100.0% |

| **Region * Head of household Crosstabulation** | | | | | | |
| --- | --- | --- | --- | --- | --- | --- |
|  | | | **Head of household** | | | Total |
|  |  |  | father | mother | other |  |
| Region | 3 region | Count | 774 | 70 | 9 | 853 |
|  |  | % within Region | 90.7% | 8.2% | 1.1% | 100.0% |
|  | others region | Count | 3850 | 331 | 84 | 4265 |
|  |  | % within Region | 90.3% | 7.8% | 2.0% | 100.0% |
| Total | | Count | 4624 | 401 | 93 | 5118 |
|  |  | % within Region | 90.3% | 7.8% | 1.8% | 100.0% |

| **Region * father.education Crosstabulation** | | | | | | | | | | |
| --- | --- | --- | --- | --- | --- | --- | --- | --- | --- | --- |
|  | | | father.education | | | | | | | Total |
|  |  |  | Illiterate | Basic literacy | Primary school | middle school | diploma | University degree | Seminary education |  |
| Region | 3 region | Count | 149 | 132 | 188 | 119 | 153 | 66 | 5 | 812 |
|  |  | % within Region | 18.3% | 16.3% | 23.2% | 14.7% | 18.8% | 8.1% | .6% | 100.0% |
|  | others region | Count | 579 | 488 | 906 | 669 | 813 | 537 | 31 | 4023 |
|  |  | % within Region | 14.4% | 12.1% | 22.5% | 16.6% | 20.2% | 13.3% | .8% | 100.0% |
| Total | | Count | 728 | 620 | 1094 | 788 | 966 | 603 | 36 | 4835 |
|  |  | % within Region | 15.1% | 12.8% | 22.6% | 16.3% | 20.0% | 12.5% | .7% | 100.0% |

| **Region * mother.education Crosstabulation** | | | | | | | | | |
| --- | --- | --- | --- | --- | --- | --- | --- | --- | --- |
|  | | | mother.education | | | | | | Total |
|  |  |  | Illiterate | Basic literacy | Primary school | middle school | diploma | University degree |  |
| Region | 3 region | Count | 281 | 140 | 172 | 93 | 113 | 49 | 850 |
|  |  | % within Region | 33.1% | 16.5% | 20.2% | 10.9% | 13.3% | 5.8% | 100.0% |
|  | others region | Count | 1041 | 719 | 908 | 476 | 739 | 342 | 4232 |
|  |  | % within Region | 24.6% | 17.0% | 21.5% | 11.2% | 17.5% | 8.1% | 100.0% |
| Total | | Count | 1322 | 859 | 1080 | 569 | 852 | 391 | 5082 |
|  |  | % within Region | 26.0% | 16.9% | 21.3% | 11.2% | 16.8% | 7.7% | 100.0% |
